# Decreased SATB1 expression promotes AML cell proliferation through NF-κB activation

**DOI:** 10.1186/s12935-019-0850-x

**Published:** 2019-05-17

**Authors:** Xiaodan Luo, Lihua Xu, Xiaohong Wu, Huo Tan, Lian Liu

**Affiliations:** Department of Hematology, First Affiliated Hospital, Guangzhou Medical University, Guangzhou, 510230 China

**Keywords:** SATB1, Acute myeloid leukemia, Gene expression profiling, RNA interference, NF-κB

## Abstract

**Background:**

Special AT-rich sequence-binding protein 1 (SATB1) is a chromatin-remodeling protein that regulates gene expressions in different types of cancer. Up-regulation of SATB1 is linked with progression of tumors. Our previous study showed that SATB1 expression was decreased in T cell leukemia/lymphoma. The contrary roles of SATB1 in solid organ tumors and hematology malignancy may provide hints to study the function of SATB1.

**Methods:**

To characterize SATB1 mRNA and protein expression in acute myeloid leukemia (AML), we performed qRT-PCR and Western blot on bone marrow mononuclear cells from 52 newly diagnosed AML patients. Stable HL-60 cell lines with knockdown of SATB1 by shRNAs sequences (HL-60 SATB1-shRNA1 and HL-60 SATB1-shRNA2) were established. Cell proliferation, cell cycle and cell invasiveness were analyzed. Murine model was established using HL-60 SATB1-shRNAs treated nude mice and tumorigenicity was compared to study the role of SATB1 in vivo. Global gene expression profiles were analyzed in HL-60 cells with SATB1 knockdown to investigate the mechanisms underlying the regulation of AML cell growth by SATB1.

**Results:**

We found that SATB1 expression was significantly decreased in patients with AML compared to normal control, and was increased after complete remission of AML. Knockdown of SATB1 enhanced the proliferation of HL-60 cells and accelerated S phase entry in vitro, and promoted the tumor growth in vivo. Global gene expression profiles were analyzed in HL-60 cells with SATB1 knockdown and the differentially expressed genes were involved in NF-κB, MAPK and PI3 K/Akt signaling pathways. Nuclear NF-κB p65 levels were significantly increased in SATB1 depleted HL-60 cells.

**Conclusions:**

Decreased SATB1 expression promotes AML cell proliferation through NF-κB activation. SATB1 could be a predictor for better response to treatment in AML.

## Background

Acute myeloid leukemia (AML) is a clinically and genetically heterogeneous disease that is fatal for over 80% of patients, particularly for those older than 60 years of age, who have less than 5% to 10% 5-year survival [1–3]. Unlike the booming development of therapies for lymphoid malignancies, myeloid drug development lagged behind due to less progress in understanding the genetic basis and pathophysiology of AML. For years, recurrent genetic abnormalities or some genetic mutations were found to clearly interact to drive the initiation, progression and relapse of AML. For example, AML with mutations of FLT3-ITD, c-Kit, TP53, etc. often has poor response to chemotherapy, high relapse rate and aggressive progression [2–8]. Chemotherapy for AML remained largely unchanged for decades, clinical response of targeted therapy is limited and the survival improvements over time were attributable mostly to hematopoietic stem cell transplantation (HSCT) [2, 5, 9]. New chromosomal aberrations, epigenetic changes and mutations of AML continue to be reported, and risk stratification is now based on genetic, genomic, and molecular characteristics which help determine the effect on prognosis, as well as provide new insights into the mechanisms of AML.

Special AT-rich sequence-binding protein 1(SATB1) is a chromatin-remodeling protein that has been shown to play an important role in tumor progression and metastasis. Aberrant expression of SATB1 was reported in various types of cancers, including laryngeal squamous cell carcinoma, gastric cancer and breast cancer [10, 11]. High SATB1 expression is usually correlated with high metastatic potential and poor prognosis of cancer [12–17]. Contrary to observations in solid tumors, SATB1 appears to suppress the progression of leukemia and lymphoma. SATB1 deficiency causes severe immunodeficiency and multiple defects in T-lineage development [18–23]. We previously reported that SATB1 expressions were decreased in T cell leukemia/lymphoma (T-ALL), Knockdown of *SATB1* significantly enhanced invasiveness of Jurkat cell in vitro [24]. It is reported that SATB1 binding to the enhancer of Sfpi1 resulted in reduced PU.1 expression in myeloid progenitor cells, which indicated that SATB1 dysfunction is associated with a subset of human AML patients [25]. However, less is known about the association between SATB1 and AML, whether SATB1 is implicated in the development of AML warrants further exploration.

In this study, we investigated the role of SATB1 in AML. We determined differences in *SATB1* expression of initial AML and the control samples, paired initial and complete remission (CR) samples to find whether SATB1 played a role in the development of AML. We found that *SATB1* expression was decreased in AML patients and increased after these patients received CR. We further studied the roles of SATB1 on AML in vitro and in vivo by shRNAs mediated knockdown of *SATB1*. We showed that knockdown of *SATB1* enhanced the proliferation of AML cells and accelerated S phase entry in vitro and promote tumorigenicity in vivo. We then analyzed the global gene expression profiles to investigate the mechanisms underlying the regulation of AML cell growth by SATB1 (part of the results were published as abstract in the 59th ASH annual Meeting: Blood 2017 130:1238).

## Methods

### Patients and clinical characteristics

Seventy-three AML patients from Oct 2015 to Jun 2017 and 38 healthy donors as control were enrolled in this study. Characteristics of newly diagnosed patients are described in Table 1. Peripheral blood samples from AML patients were obtained at initial diagnosis (N = 52) with informed consent from patients or their legal guardians. All initial samples were obtained before any anti-leukemia treatment was given. The study was approved by the Committee on the Ethics of the First Affiliated Hospital of Guangzhou Medical University (Permit number: 2012-41).

**Table 1:** Patient Characteristics

Variable	*SATB1*_low_ (N = 32)		*SATB1*_high_ (N = 20)		*P* value
	Median (range)	No.	Median (range)	No.	
Age, years	50.5 (19–78)		51 (16–79)		0.839
≤ 55		19		12	
> 55		13		8	
Gender					0.430
Male		18		9	
Female		14		11	
WBC (× 10^9^)	16.82 (0.7–198.34)		11.32 (0.6–283.8)		0.549
RBC (× 10^12^)	2.4 (1.33–4.49)		2.715 (1.25–4.04)		0.706
HGB (g/L)	72 (49–120)		83.5 (40–129)		0.600
PLT (× 10^9^)	47 (3–97)		56 (8–104)		0.624
Blast (%)	60 (19.7–100)		71.25 (22–94)		0.420
FAB subtype					
M0		1		0	
M1		2		3	
M2		9		6	
M3		6		3	
M4		5		3	
M4Eo		1		0	
M5		6		4	
Mixed AML		2		1	
CR rate		57.14%		76.47%	0.189
Missing value^a^		4		3	

WBC, white blood cell; RBC, red blood cell; HGB, hemoglobin; PLT, platelet; CR, complete remission

^a^Four patients refused the treatment after diagnosed and three patients died during the first cycle of chemotherapy

### Mice

BALB/c (H-2d, n = 12) nude mice were purchased from Animal experimental center, Guangdong, China. Nude mice were housed and maintained in a specific pathogen-free environment and used when they were between 4 and 5 weeks of age. The protocol was approved by the Committee on the Ethics of Animal experimental center, Guangdong, China (Permit number: SYXK 2016-0168). HL-60 *SATB1*-shRNA1 and HL-60 control cells (1 × 10^7^) were injected subcutaneously in *SATB1*-shRNA1 and control mice, respectively. Tumor size was measured everyday and tumor volume was calculated by the formula: tumor volume [mm^3^] = (length [mm]) × (width [mm])^2^ × 0.5 [26].

### Cell lines

HL-60, HEL, THP-1 cells (Cell Bank, Chinese Academy of Sciences) and NB4 cells (Cell Resource Center, IBMS, CAMS/PUMC) were cultured in RPMI-1640 medium supplemented with 10% fetal bovine serum (FBS; Hyclone, Logan, UT), penicillin (100 units/ml), and streptomycin (100 units/ml) maintained at 37 °C in humidified 5% CO_2_ incubator.

### Plasmid Construction and Retroviral Infection

After synthesis of the oligoduplexes, the GV248.puro plasmid vector was digested with AgeI and EcoRI restriction endonucleases. Next, the *SATB1*-specific shRNAs were cloned into the GV248.puro vector. The successful plasmid construction was verified by DNA sequencing. Production of lentivirus was performed according to the instructions; HL-60 cells were subjected with infection of lentivirus expressing *SATB1*-shRNA1 and *SATB1*-shRNA2 or GV248 (empty vector). HL-60 *SATB1*-shRNA and HL-60-CTR cells were cultured with puromycin for 3 days to produce stable *SATB1* knockdown cell line. Targeted *SATB1* sequence is as follows: *SATB1*-shRNA1, GGATTTGGAAGAGAGTGTC; and *SATB1*-shRNA2, GTCCACCTTGTCTTCTCTC.

### RNA extraction and quantitative real-time PCR assays (qRT-PCR)

Total RNA was extracted using TRIzol reagent (Invitrogen). Complementary DNA (cDNA) was synthesized from 1 μg of total RNA according to RevertAid™ First-Strand cDNA Synthesis Kit #K1622 instruction (Thermo Scientific). cDNAs were subjected to qRT-PCR analysis with specific primers. qRT-PCR was performed using a FQD-48A (M2) instrument (BIOER, China) with iQTM SYBR Green supermix (Bio-Rad). All reactions were run in triplicate in three independent experiments and amplified in a 10 μl reaction according to the manufacturer’s protocol. Cycling conditions included an initial hold step (95 °C for 30 s) and 40 cycles of a two-step PCR (95 °C for 30 s and then 53 °C for 30 s), followed by a dissociation step (95 °C for 15 s, 60 °C for 30 s, and then a sequential increase to 95 °C). Relative messenger RNA (mRNA) expression was calculated by the comparative 2^−ΔΔCT^ method [27]. The primers were: SATB1 (Forward: 5′-TGCAAAGGTTGCAGCAACCAAAAGC -3′; Reverse: 5′-AACATGGATAATGTGGGGCGGCCT-3′), GAPDH (Forward: 5′- TGTTG CCATCAATGACCCCTT-3′; Reverse: 5′-CTCCACGACGTAC TCAGCG-3′); GAPDH was used as an internal control.

### Cell Proliferation assay

HL-60 *SATB1*-shRNA1 cells, HL-60 *SATB1*-shRNA2 cells and HL-60 CTR cells were planted at a density of 2000 cells/well in 96-well plates and maintained at 37 °C in humidified 5% CO2 incubator for 24 h. 10 µl of the Cell Counting Kit-8 (CCK8, Dojindo, Japan) solution was added per well and the plate was incubated for 4 h, the absorbance at 450 nm was measured on a microplate reader.

### Cell invasion assay

The invasive potential of HL-60 *SATB1*-shRNA1 cells, HL-60 *SATB1*-shRNA2 cells was examined using transwell inserts fitted with polycarbonate filters (8-μm pore size, Costar, Cambridge, MA) coated with matrigel (BD Biosciences, Bedford, MA). Matrix solutions within transwells were polymerized at 37 °C for 1 h and dried onto the transwells overnight at room temperature. Cells were seeded in the upper compartment without FBS medium while lower wells contained 10% FBS medium. After 48 h of incubation, the cells in the upper chamber were removed while other cells, which had passed through the filter, were fixed with 3.7% paraformaldehyde, stained with 0.2% crystal violet, counted, and captured at ×100 and ×400 magnifications using the camera on inverted microscope. Contents of the lower compartments were collected, and migrated cells were also counted.

### Cell Cycle Analysis

Cell cycle distribution of HL-60 *SATB1*-shRNA1 cells, HL-60 *SATB1*-shRNA2 cells and HL-60 CTR cells was determined by flow cytometric analysis. Cells were re-suspended into 5 × 10^5^ cells/ml and were collected for nuclear staining which was performed according to the manufacturer’s instructions using Flow Cytometry Analysis of Cell Cycle Kit (GENMED, Shanghai). Following staining, cells were immediately analyzed by flow cytometry.

### Nuclear and Cytoplasmic Proteins Extraction

The nuclear and cytoplasmic proteins were extracted using an Nuclear and Cytoplasmic Protein Extraction Kit (Keygen, Shanghai, China) according to the manufacturer’s instructions. Cells were harvest and washed with ice-cold PBS. Supernatant was carefully remove and discard. The cells were mixed with Buffer A and Buffer B and the tube was incubated on ice for 30 min. Vortex the tube at 3000×*g* for 10 min to fully suspend the cell pellet. Immediately transfer the supernatant (cytoplasmic extract) to a clean pre-chilled tube. Suspend the insoluble fraction in Buffer C. Vortex on the highest setting for 15 s. Place the sample on ice for 30 min and continue vortexing for 15 s every 10 min, for a total of 40 min. Centrifuge the tube at 14,000×*g* in a microcentrifuge for 30 min. Immediately transfer the supernatant (nuclear extract) fraction to a clean pre-chilled tube. Store extracts at − 80 °C until use. The protein concentrations were determined using a BCA protein assay according to the manufacturer’s instructions.

### Western blotting

Expressions of SATB1 were detected in HL-60, NB4, HEL and THP-1 cells and compared in HL-60 SATB1-shRNA1 cells, HL-60 SATB1-shRNA2 cells and HL-60 CTR cells using western blotting. Expressions of p38 MAPK, phospho-p38 MAPK, Akt, phospho-Akt, total NF-κB p65, nuclear NF-κB p65, plasma NF-κB p65 were also analyzed in HL-60 SATB1-shRNA cells and HL-60 CTR cells using western blotting according to the standard procedure [28]. Rabbit anti-SATB1 primary antibodies (1:3500 dilution) and horseradish-peroxidase conjugated anti-rabbit IgG secondary antibody (1:3000 dilution) were used.

### Gene expression profiling and the KEGG pathway analysis

Integrity of total RNA was checked using the Agilent 2100 Bio-analyzer (Agilent, Santa Clara, USA). Gene expression profiling was performed by the Dana-Farber Microarray Core Facility using Affymetrix U133 Plus 2.0 chip (Affymetrix, Santa Clara, USA) following the manufacturer’s guidelines, and data was analyzed by DAVID software. Gene expression was compared between HL-60 *SATB1*-shRNA1 cells and HL-60 CTR cells and analyzed by KEGG Pathway. Genes that displayed two-fold up-regulation or 0.5-fold down-regulation in expression in comparisons were selected for further analyses. Arrays with poor quality according to the manufacturer’s recommendations were excluded from further analysis.

### Statistical analysis

The data were reported as mean ± S.D. Differences among three groups were determined by analysis of Mann–Whitney U, whereas differences between two groups were evaluated by the Student’s *t* test. Chi square test was done to study the association of SATB1 expression with multiple factors. P values less than 0.05 were considered statistically significant. Statistical analysis was performed by the SPSS 19.0 (SPSS Inc., Chicago, USA) statistical software programs.

## Results

### SATB1 expression was decreased in AML patients

**Fig. 1:**
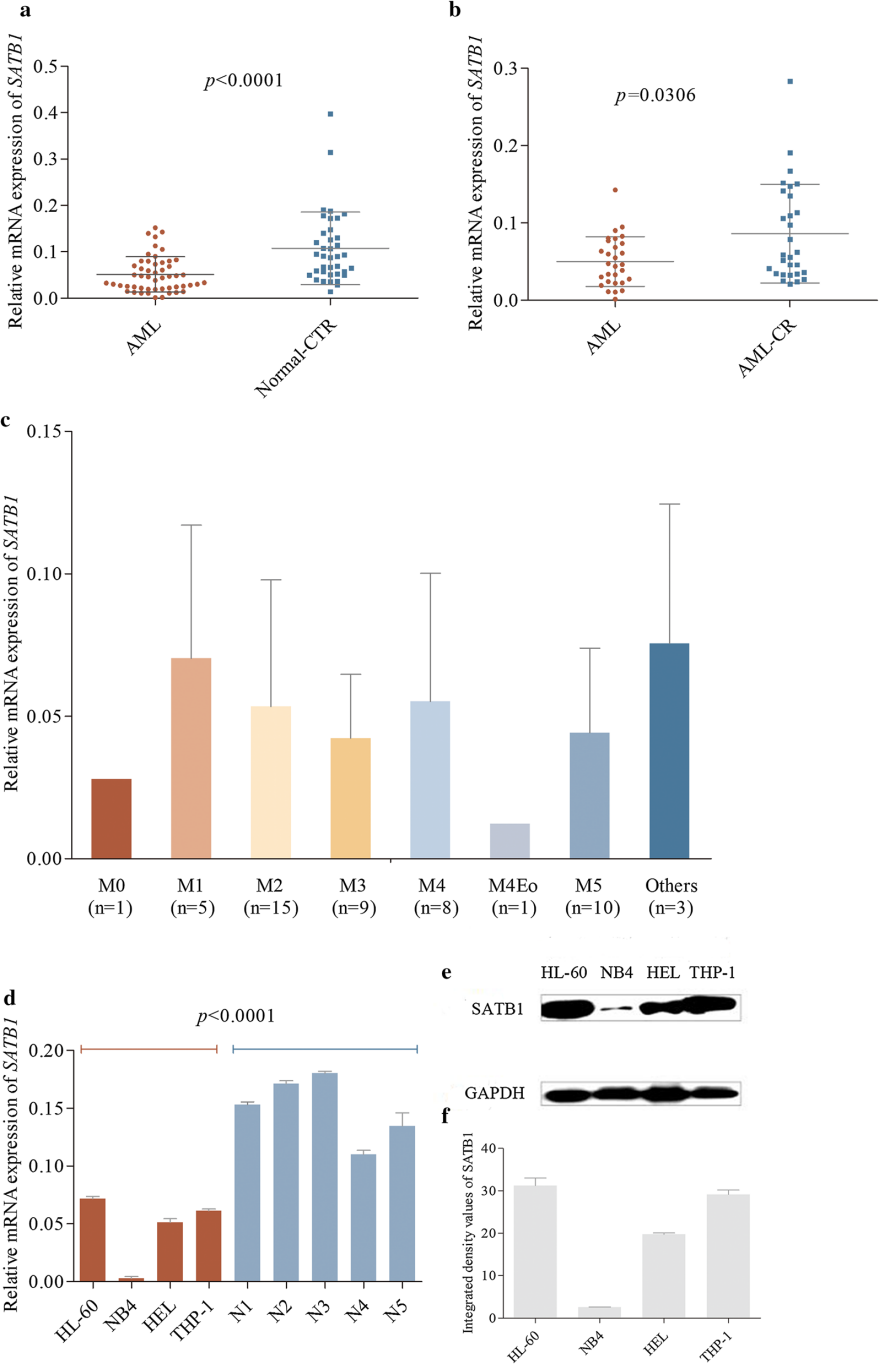
*SATB1* expression was decreased both in newly diagnosed AML patients and AML cell lines compared to healthy donors, but increased after patients achieved CR. **a** *SATB1* mRNA expression was lower in 52 newly diagnosed AML patients compared to 38 healthy donors (*P *< 0.0001). **b** *SATB1* mRNA expression was increased in 29 CR samples compared to their initial AML samples (*P *= 0.0306). **c** *SATB1* expression in AML based on FAB classification including 3 cases of mixed AML. **d** *SATB1* mRNA expression was lower in 4 AML cell lines: HL-60, NB4, HEL and THP-1 (*P *< 0.0001). **e**, **f** Western blotting showed that SATB1 exhibited highest expression in HL-60 cells among 4 AML cell lines (*P *< 0.0001)

To evaluate the association between SATB1 and AML, *SATB1* expression was detected in 52 newly diagnosed AML patients and the normal control. Results showed that *SATB1* mRNA expression was significantly lower in AML samples (*P *< 0.0001, Fig. 1a). In 52 newly diagnosed AML patients, 29 patients received CR after one or two cycles of chemotherapy. We then evaluated the correlations between SATB1 and AML status. SATB1 expression was increased in CR patients compared to their initial samples (*P *= 0.0306, Fig. 1b), indicating that SATB1 could be a predictor for better response to treatment. According to the average expression of *SATB1* of 52 AML patients, patients were then categorized into *SATB1*_high_ and *SATB1*_low_, of which the CR rate was 76.47% and 56.14%, respectively (*P *= 0.1891, Table 1). Next, we analyzed the correlation between SATB1 expression and FAB category to assess whether low SATB1 expression is indicated in some specific subtypes of AML with poor clinical outcome. We observed no difference among FAB subtypes of AML regarding SATB1 expression which also has not been related to age, sex, blood cell count and percentage of blast (Fig. 1c).

### Knockdown of SATB1 enhanced the proliferation of AML cells

**Fig. 2:**
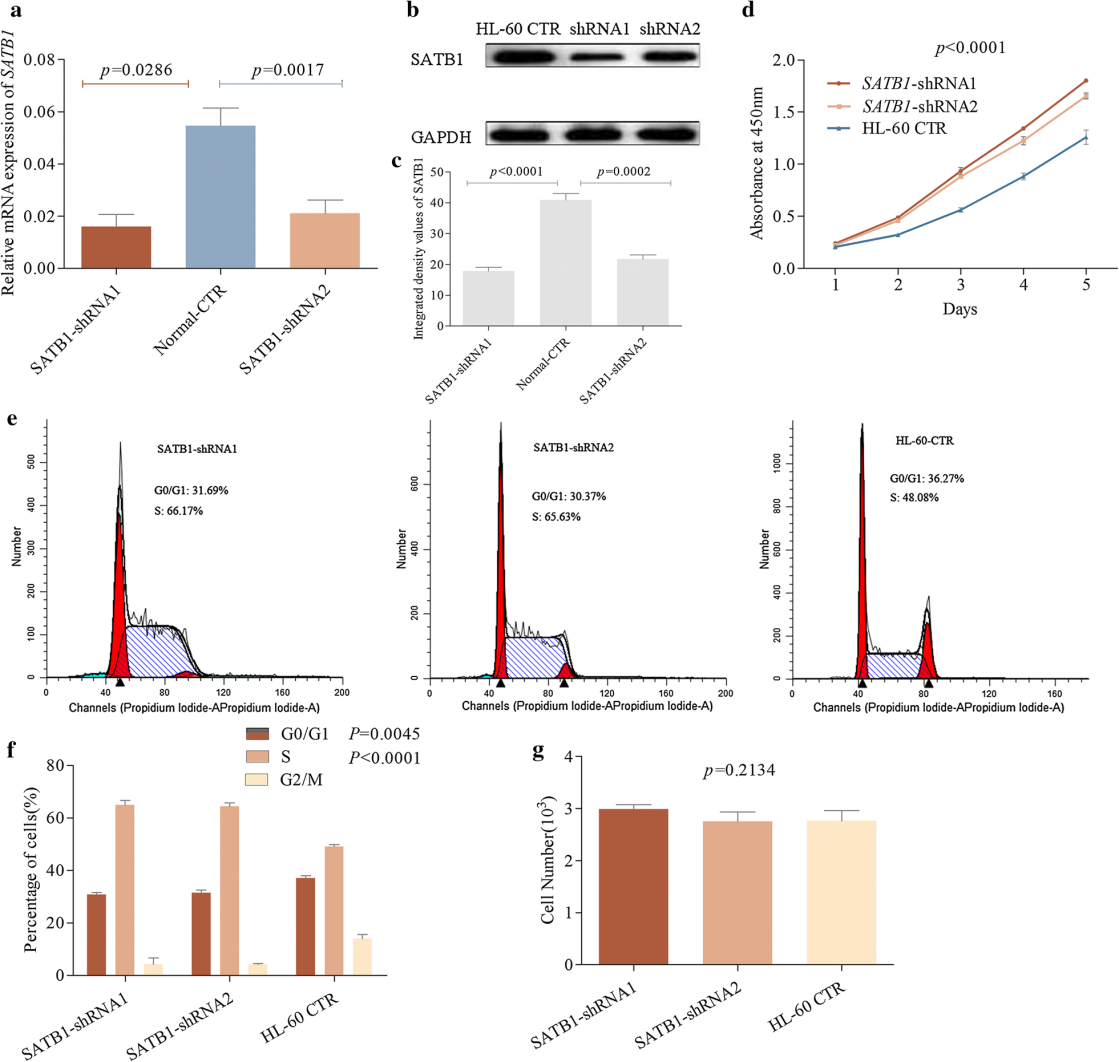
*SATB1* knockdown led to a progressive increase in the proliferation of HL-60 cells and accelerated S phase entry, but did not increase the invasiveness of AML cells. **a** *SATB1* mRNA expression was decreased in HL-60 *SATB1*-shRNA1 and *SATB1*-shRNA2 cells (*P *= 0.0286, *P *= 0.0017). **b**, **c** Decreased SATB1 protein levels were confirmed by western blotting (*P *< 0.0001, *P* = 0.0002). **d** CCK assay was performed and the absorbance at 450 nm was measured. Increased proliferation of HL-60 cells was detected from day 2 to day 5. **e** Knockdown of *SATB1* Cell cycle was analyzed by flow cytometry in *SATB1*-shRNA1, 2 and HL-60 CTR group. **f** The percentage of cells in *SATB1*-shRNA1/2 was reduced in the G0/G1 phase but increased in S phase (*P *= 0.0045, *P *< 0.0001). **g** Cell Invasion was analyzed by transwell assay and the result showed that down regulation of *SATB1* did not increase the penetration of AML cells through the matrigel-coated membrane (*P *= 0.2134)

To define the association between *SATB1* expression and the development of AML, we evaluated SATB1 expression in HL-60, NB4, HEL, THP-1 cells and samples from healthy donors as control. Results showed that *SATB1* expression was decreased in all AML cell lines than in control (*P *< 0.0001, Fig. 1d). SATB1 exhibited highest expression in HL-60 cells among 4 different cell lines (*P *< 0.0001, Fig. 1e, f), we investigated its role in HL-60 by shRNAs mediated knockdown of *SATB1*. As shown in Fig. 2a, b, C HL-60 *SATB1*-shRNA1 and *SATB1*-shRNA2 cells exhibited decreased SATB1 expression compared to the control (HL-60-CTR) both in the mRNA (*P *= 0.0286, *P *= 0.0017) and protein levels (*P *< 0.0001, *P* = 0.0002), suggesting that the stable *SATB1* knockdown cells were successfully established. To define the role of SATB1 in HL-60 cells, we performed CCK8 assay to evaluate the proliferation of HL-60 *SATB1*-shRNA cells. *SATB1* knockdown led to a progressive increase in the proliferation of HL-60 cells both in *SATB1*-shRNA1 and *SATB1*-shRNA2 groups since day2 (*P *< 0.0001, Fig. 2d).

### Down regulation of SATB1 accelerated S phase entry but did not increase the invasiveness of AML cells

To study the effect of SATB1 on AML cell cycle, we compared the number of quiescent and actively cycling HL-60 cells in *SATB1* shRNA1, *SATB1* shRNA2 and CTR group using flow cytometry. The number of quiescent cells in the G0/G1 phase of the cell cycle was significantly reduced in the absence of *SATB1* (*P *= 0.0045). Consistently, the number of cells in S phase was increased (*P *< 0.0001), which suggested that SATB1 promoted quiescence of AML cells while knockdown of *SATB1* enhanced cell activation (Fig. 2e, f). In order to further evaluate the impact of SATB1 on the invasiveness of AML cells, we cultured HL-60-CTR cells and HL-60 *SATB1*-shRNA cells in transwell matrigel-coated chambers under the same conditions and compare the invasiveness between both cell lines. We found that shRNA mediated knockdown of *SATB1* did not cause significant increase of penetration through the matrigel-coated membrane, which showed similar invasiveness as HL-60-CTR cells (Fig. 2g, *P *= 0.2134).

### Knockdown of SATB1 promoted tumorigenicity in nude mice

**Fig. 3:**
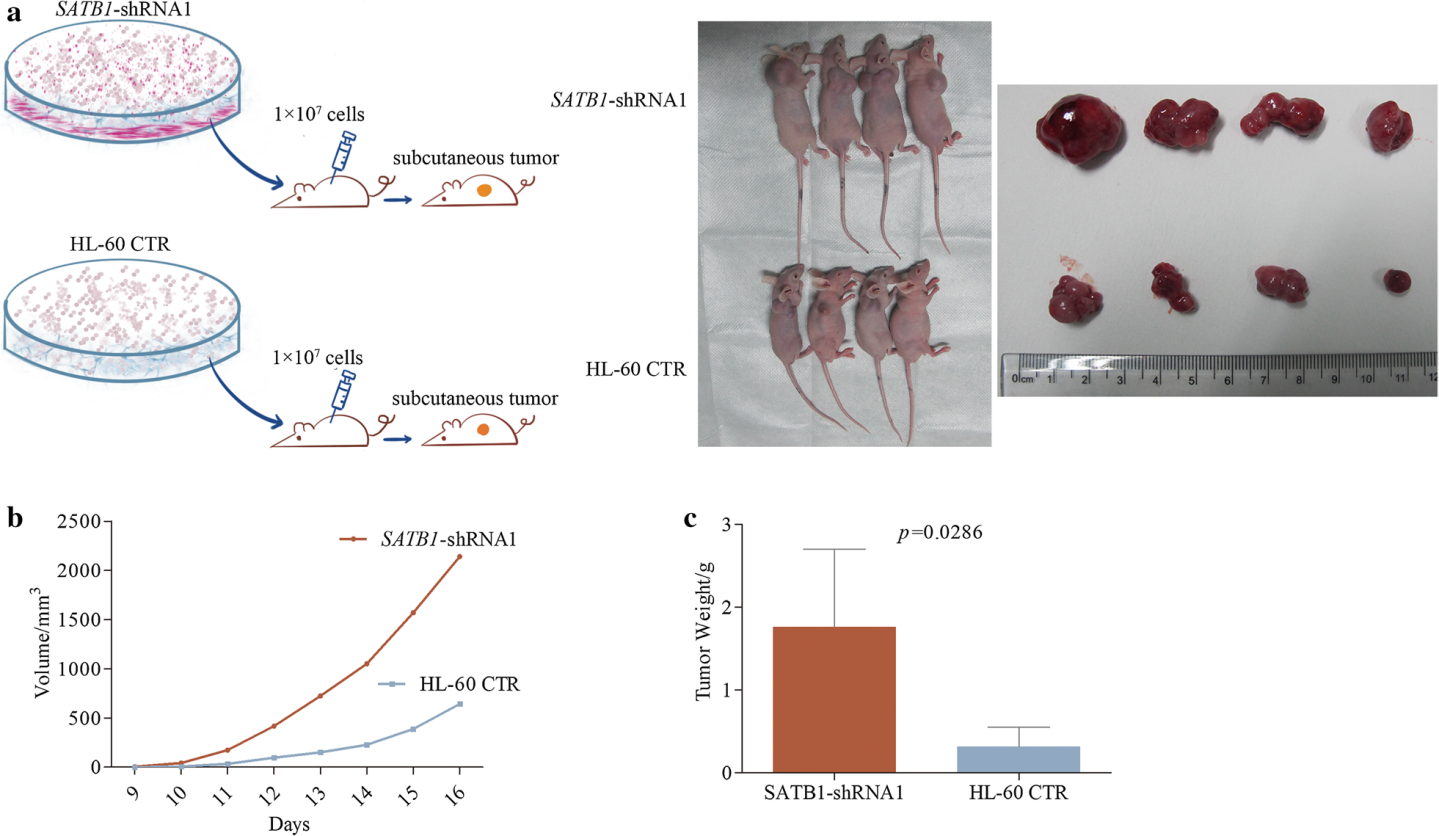
Knockdown of *SATB1* promoted tumorigenicity in nude mice. **a** HL-60 *SATB1*-shRNA1 and HL-60 CTR cells (1 × 10^7^) were injected subcutaneously in *SATB1*-shRNA1 and CTR mice, respectively. Tumor was formed in 4 mice in each group. All mice were executive 3 weeks after injection. **b** Tumor size was measured everyday and the result showed that tumor volume of *SATB1*-shRNA1 was larger compared to CTR mice. **c** Tumor weight showed a significant increase in *SATB1* shRNA mice (*P *= 0.0286)

To further test if *SATB1* knockdown could promote AML progression in vivo, we compared the tumor size and weight in HL-60 *SATB1*-shRNA1 cells treated mice and the CTR mice. Mice were injected subcutaneously with 1 × 10^7^ HL-60 *SATB1*-shRNA1 cells (N = 6)) or HL-60 cells (CTR, N = 6) and all mice were executive 3 weeks after injection (Fig. 3a). Tumor was formed in 4 *SATB1*-shRNA1 mice and 4 CTR mice at the average time of 9 ± 1 day and 10 ± 1 day, respectively. Tumor size was compared from day 9 to day 16 and *SATB1* shRNA mice showed a larger tumor volume compared to CTR mice (Fig. 3b). At the end of the experiment, tumor weight was measured and showed a significant increase in *SATB1* shRNA mice compared to CTR mice (*P *= 0.0286, Fig. 3c).

### SATB1 regulated AML cell growth via the activation of NF-κB signaling pathway

**Fig. 4:**
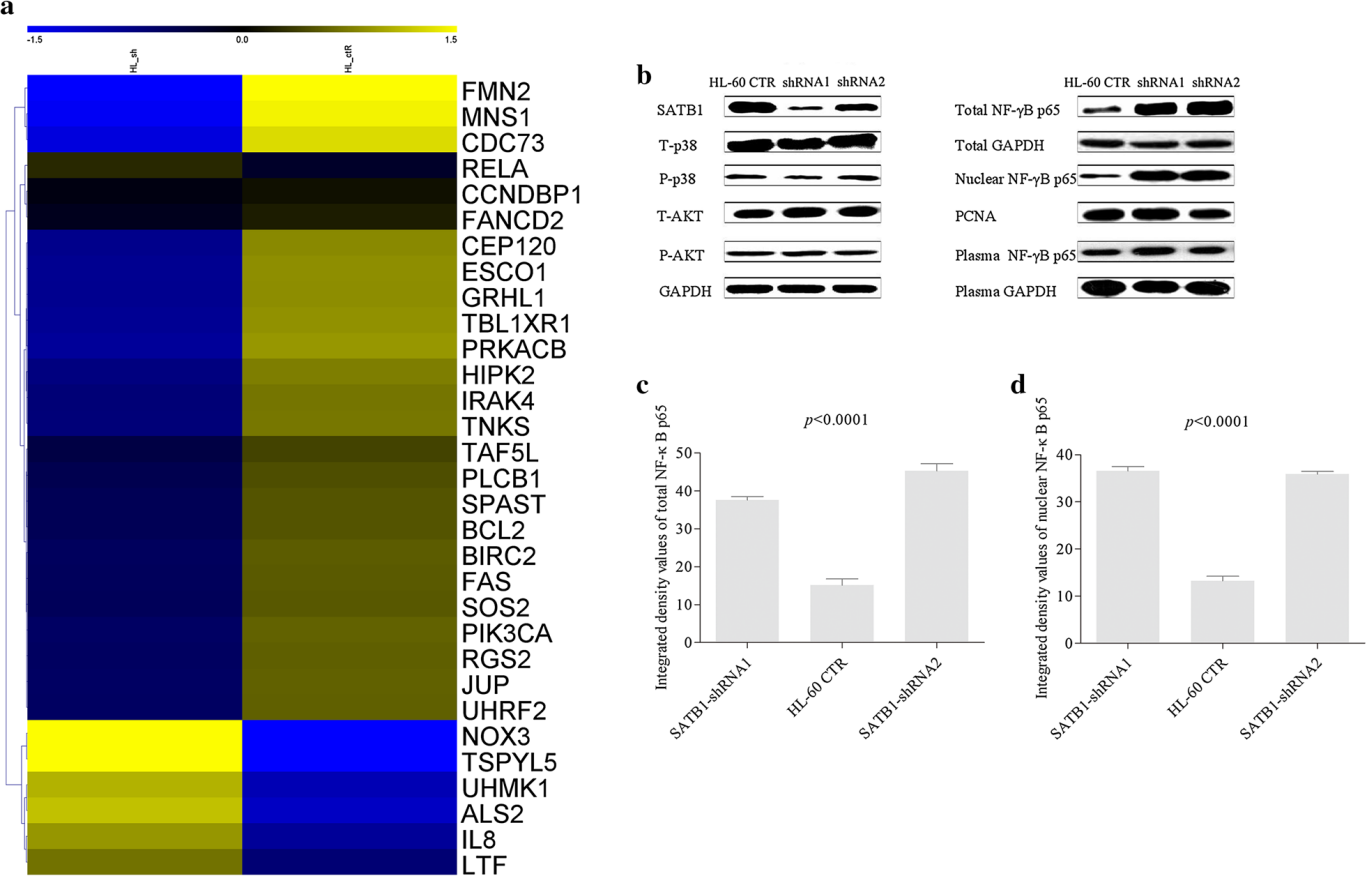
SATB1 regulated AML cell growth via the activation of NF-κB signaling pathway. Gene expression was compared between *SATB1*-shRNA1 cells and HL-60 CTR cells and the results were analyzed by KEGG Pathway. **a** The hot map showed 862 downregulated and 377 upregulated genes in *SATB1*-shRNA cells. **b** Protein levels of p38 MAPK, phospho-p38 MAPK, Akt, phospho-Akt and total NF-κB p65 in both *SATB1*-shRNA and CTR cells were assessed by western blotting. **c**, **d** Results showed that total and nuclear NF-κB p65 levels were significantly increased in *SATB1*-shRNA cells, while cytoplasm protein levels showed no difference (*P *< 0.0001)

To investigate the mechanisms underlying the enhanced proliferation of HL-60 *SATB1*-shRNA cells, we analyzed the global gene expression profiles of HL-60 *SATB1*-shRNA cells and HL-60-CTR cells. Genes were identified as differentially expressed if their expression showed at least two-fold difference in each of the comparisons. Using these criteria, we identified 862 downregulated and 377 upregulated genes in HL-60 *SATB1*-shRNA cells (Fig. 4a). Downregulated genes were involved in the regulation of cell cycle, proliferation, apoptosis and cell death. Upregulated genes were involved in chromatin assembly and disassembly, protein-DNA complexes assembly, cellular cation homeostasis and inflammatory response. KEGG based analysis was used to determine whether particular biological pathways gene expression was more highly represented in HL-60 *SATB1*-shRNA cells versus HL-60-CTR cells. Differentially expressed genes were involved in NF-κB, MAPK and PI3 K/Akt signaling pathways. We therefore assessed the protein levels of p38 MAPK, phospho-p38 MAPK, Akt, phospho-Akt and total NF-κB p65 in both *SATB1*-shRNA and CTR cells. Results showed that total NF-κB p65 levels were significantly increased in HL-60 *SATB1*-shRNA cells (Fig. 4b, c). Since nuclear and cytoplasm NF-κB p65 play different roles in cell proliferation, we next compared both nuclear and cytoplasm NF-κB p65 levels among groups. Nuclear NF-κB p65 levels were significantly increased in HL-60 *SATB1*-shRNA cells, while cytoplasm protein levels showed no difference (Fig. 4d). Together, these results indicate that shRNA mediated knockdown of *SATB1* regulated AML cell growth via the activation of NF-κB signaling pathway.

## Discussions

SATB1 is a global genomic organizer that participates in activation and inactivation of genes and the differentiation of embryonic stem cells, thus is implicated in a variety of cancers and cancer progression [4, 5, 15, 17, 22, 29–31]. Up-regulation of *SATB1* was found in many epithelial tumors and negatively correlated with the prognostic properties and clinical outcome [6, 15, 32]. Recent studies in cells with SATB1 knockdown have emphasized the functional relevance of SATB1 to carcinogenesis [17, 32, 33]. The mechanism by which SATB1 regulate cancer development remains unclear and has been extensively studied. It’s reported that SATB1 might cooperatively regulate expression of the anti-apoptotic BCL2 and pro-apoptotic NOXA genes to participate in disruption of apoptosis, which is a hallmark of cancer that reinforces tumorigenesis and resistance to cytotoxic cancer therapies [6, 34]. However, *SATB1* expression was decreased rather than increased in hematology malignancy. We previously reported that SATB1 expressions were up-regulated in T-ALL. Knockdown of *SATB1* have enhanced invasive potential in Jurkat cells [24]. These observations raised interesting questions of whether SATB1 play the similar role in AML as in T-ALL or in epithelial tumors and whether it could be one of the potential treatment targets for AML. In this study, our data showed that SATB1 expression was deregulated in 52 initial AML samples, which is in line with our own findings on T-ALL. By comparing SATB1 expression of pair initial AML and CR patients, we found that SATB1 expression was increased in CR patients. Although the CR rate was not significantly increased as expected as SATB1 expression was up-regulated, it’s higher in *SATB1*_high_ patients and the difference could be statistical significant if the sample size is larger. We then confirmed decreased SATB1 expression in different AML cell lines and found that SATB1 was more specific in HL-60 cells. These data suggested that down-regulation of SATB1 could be an important factor in leukemogenesis.

SATB1 is predominantly expressed in the thymus and highly associated with thymocyte differentiation. It is thought to induce lymphopoiesis and regulate the expression of various genes during lymphocyte especially T cell development, so SATB1 is thought to be correlated to the development of ALL [18–22, 28, 31, 35–40]. SATB1 regulates many genes involved in HSC lineage decisions and development, but evidence that supports the role of SATB1 in AML remained elusive [41, 42]. It is reported that SATB1-deficient HSCs were found to be less quiescent and more prone to differentiate to myeloid–erythroid lineages, which indicated that SATB1 is associated with the regulation of myeloid lineages and its dysfunction could be linked to AML [1–6, 25, 29].

One aim of this study was to describe the association between SATB1 and AML. *SATB1* was knockdown in the selected HL-60 cell line. We observed that knockdown of *SATB1* resulted in a progressive proliferation of HL-60 cells. HL-60 *SATB1*-shRNA cells were then injected subcutaneously in mice. As expected, progression of leukemia was proved by significant increased tumor weight, suggesting that *SATB1* knockdown could increase AML proliferation both in vitro and in vivo. In addition, cell cycle analysis was then performed and we showed that down regulation of *SATB1* could promote cell activation by accelerating S phase entry. These results suggest an indispensable role of SATB1 in the development of AML. However, we did not observe a significant increased in the invasiveness of AML cells after knockdown of *SATB1*. It is difficult to make a conclusion that SATB1 is dispensable for the invasiveness of AML because AML arise as a consequence of a number of genetic aberrations and multiple molecular events; the invasiveness of different subtypes of AML varied and results from only one AML cell line can not tell the whole story. More detailed analysis is necessary for the mechanisms underlying the enhanced proliferation of AML cells by SATB1.

SATB1 regulates multiple genes in lymphocyte differentiation. It is reported that SATB1 interacted with β-catenin which acts as a transcriptional regulator mediating Wnt signaling thus inducing target gene expressions [24, 36, 43]. SATB1 also associate with epigenetic modifiers such as histone deacetylase which is important for lymphocyte development [20]. However, what target genes and the associated signal pathways are possibly involved in the regulation of AML by SATB1has not been clarified. In this study, we compared the global gene expression profiles of HL-60 *SATB1*-shRNA cells and HL-60-CTR cells. For those differentially expressed genes which were involved in NF-κB, MAPK and PI3K/Akt signaling pathways, we assessed the protein levels and found that nuclear NF-κB p65 levels were significantly increased in HL-60 *SATB1*-shRNA cells, while cytoplasm protein levels showed no difference when compared to HL-60-CTR cells. These findings suggested that knockdown of *SATB1* might regulate AML cell growth via the activation of NF-κB signaling pathway.

## Conclusions

We demonstrated in this study that impairment of SATB1 is associated with AML. Knockdown of *SATB1* promoted cell proliferation and tumorigenicity of AML via the activation of NF-κB signaling pathway. Further study about gene regulatory network modulated by SATB1 may lead to the development of a new treatment of AML.

## Data Availability

The datasets used and/or analyzed during the current study are available from the corresponding author upon reasonable request.

## Abbreviations

SATB1: special AT-rich sequence-binding protein 1
AML: acute myeloid leukemia
HSCT: hematopoietic stem cell transplantation
T-ALL: T cell leukemia/lymphoma
CR: complete remission
CCK8: Cell Counting Kit-8
